# Changes in numbers and size of synaptic vesicles of cortical neurons induced by exposure to 835 MHz radiofrequency-electromagnetic field

**DOI:** 10.1371/journal.pone.0186416

**Published:** 2017-10-18

**Authors:** Ju Hwan Kim, Hyo-Jeong Kim, Da-Hyeon Yu, Hee-Seok Kweon, Yang Hoon Huh, Hak Rim Kim

**Affiliations:** 1 Department of Pharmacology, College of Medicine, Dankook University, Cheonan, Chungnam, South Korea; 2 Center for Electron Microscopy Research, Korea Basic Science Institute, Ochang, Chungbuk, South Korea; University of Cincinnati College of Medicine, UNITED STATES

## Abstract

We studied the effects of radiofrequency electromagnetic fields (RF-EMFs) exposure on neuronal functions of mice. Particularly, we focused on RF-EMF effects on synaptic vesicles (SVs), which store neurotransmitters at axon terminals or synaptic boutons. C57 BL/6 mice were exposed to 835 MHz RF-EMF (4.0 W/kg SAR, for 5 h daily) and alterations in SVs at presynaptic terminals in the cerebral cortex were determined. Ultrastructure of randomly selected cortical neurons was observed using typical electron microscopy and bio-high voltage electron microscopy (Bio-HVEM) methods, which enable the estimation of the numbers and size of SVs. The density of the SVs (number /10 μm^2^ or 40 μm^3^) was significantly decreased in the presynaptic boutons of cortical neurons after RF-EMF exposure. Furthermore, qPCR and immunoblotting analyses revealed that the expression of synapsins I/II (Syns I/II) genes and proteins were significantly decreased in the cortical neurons of RF-EMF exposed mice. The present study suggested that alteration of SVs and Syn levels may result in alterations of neurotransmitters in the cerebral cortex following RF-EMF exposure.

## Introduction

Exposure to radiofrequency electromagnetic fields (RF-EMFs) is inevitable owing to widespread usage of mobile phones and wireless communication devices in modern life. The use of these devices has raised public concerns and substantial controversy regarding the potential biological health effects of RF-EMF exposure in humans [1]. Notably, in 2011, the International Agency for Research on Cancer classified RF-EMF as a Group 2B carcinogen, and warned the public of the potential biological risks of mobile phone use [2]. In particular, mobile phone usage usually requires immediate contact with the head and implicates a close-proximity exposure and thus a high possibility of the RF-EMF affecting the brain nervous system. However, the correlation between cancer or neurological diseases and RF-EMFs is still unclear.

In the recent decade, the effects of RF-EMF have been reported not only on the permeability of the blood-brain barrier, intracellular calcium homeostasis, demyelination, and neuronal damage in animal brains [3–6] but also on various cellular processes such as apoptosis, autophagy, and extracellular signal pathways [3, 7, 8] that may result in behavioral disorders [9]. In addition, epidemiologic studies have reported cognitive dysfunctions as well as electromagnetic hypersensitivity [3, 10, 11]. Furthermore, radiofrequency electromagnetic radiations lead to decrease the number of synaptic vesicles(SVs) at the presynaptic terminal of the CA3 area in rat hippocampus [12]. Based on the results of these studies, it has been suggested that RF-EMF exposure can induce various neurological changes.

The cerebral cortex, a thin layer composed of folded bulges (gyri) and deep furrows (sulci), is situated in the outer part of the brain [13]. The cerebral cortex is a highly developed region in the human brain and plays specific roles in sensory functions, thought, memory, attention, perception, and language [14]. Dysfunctions of the cerebral cortical region in humans could be related to various neurodegenerative diseases such as Alzheimer's disease, Lafora disease, and various cognitive disorders [15–17].

In this study, we explored whether the synaptic vesicles (SVs) at cerebral cortex of C57BL/6 mice is affected by 835 MHz RF-EMF (4.0 W/kg SAR, for 5 h/day, for 4 and 12 weeks) exposure. Since SVs play important roles in the synapses by regulating neurotransmitter storage and release, morphological changes of SVs may cause alterations in neurotransmitter release. Thus, the morphological alterations of the SVs was evaluated using conventional TEM analysis for estimating the number and the size of SVs, additionally three-dimensional (3D) electron tomography with bio-high voltage electron microscopy (Bio-HVEM) for more accurate evaluation of the number of SVs at the presynaptic terminals in response to RF-EMF exposure.

In addition, to find whether any SV-related proteins were also changed in response to RF-EMF exposure, we chose synapsin (Syn) proteins, which are a multigene family of neuron-specific phosphoproteins and the key regulators of the SV life cycle [18]. Moreover, Syns are SV-associated proteins and the most abundant proteins on SVs, which tether the SVs to each other or to cytoskeleton (i.e., actin filaments) and release the SVs in presynaptic nerve terminals and are implicated in the regulation of neurotransmitter release and synapse formation [18], thereby regulating the availability of SVs for exocytosis [19]. Therefore, we further examined the expression levels of Syns in the cerebral cortex following RF-EMF exposure.

## Materials and methods

### Mice

C57BL/6 male, 6-week-old mice (weight, 25–30 g) were purchased from Daehan Bio Link (Chungbuk, South Korea) and maintained under controlled conditions (an ambient temperature of 23 ± 2°C and 12-h light/dark cycles). Food pellets (Daehan Bio Link) and water were supplied ad libitum. After a week of adaptation period, mice were randomly assigned to either sham-exposed or RF-EMF-exposed groups. All mice procedures were in accordance with the National Institutes of Health Guidelines for Animal Research and were approved by Dankook University Institutional Animal Care and Use Committee (IACUC; DKU-15-001), which adheres to the guidelines issued by the Institution of Laboratory of Animal Resources.

### RF-EMF exposure

Mice were exposed to 835 MHz RF-EMF using a Wave Exposer V20; the dosimetry (i.e. measuring spectrum (MHz) or body-average SAR value) for our RF-EMF generator has been described in detail previously [6]. Whole body exposure was at a specific absorption rate (SAR) of 4.0 W/kg for 5 h daily, for 4 and 12 weeks for the randomly allocated mice (RF). The other mice received sham treatment for certain periods. The sham-treated group was maintained under identical environmental conditions and treated in the same circular pattern as the RF-EMF exposed groups, but without RF-EMF exposure. The sham-treated and RF-EMF exposed mice could move freely in their cage. The space for the mouse cage in the RF-EMF generator was 43 cm long × 37 cm wide × 18 cm high. RF-EMF exposure was a top horn antenna to the lower mouse cage. The bottom and wall of the cage was covered with ceramic wave absorption material. The design was intended to mimic RF-EMF with SAR exposure in an open environment, to exclude the possibility of the influence of the number of mice on exposure. Importantly, the RF-EMF exposure apparatus was equipped with automatic light systems, air conditioning, and water dispensers. The mice movements in the cage were not restricted during the exposure. All the experiments were conducted in our animal facility, which was maintained at constant temperature. To rule out the possibility of thermal effect due to the RF-EMF generator, mice body temperatures were measured before and after RF-EMF exposure. The results indicated that exposure to RF-EMF of the free moving mice did not affect their body temperature during the 5 h exposure to 835 MHz RF-EMF at 4.0 W/kg SAR emitted from our RF-EMF generator [6].

### Transmission electron microscopy

Experimental mice were euthanized by cervical dislocation and head was quickly decapitated with scissors, then the cerebral cortex was rapidly dissected from each brain on ice. The cerebral cortex, dissected from mice of the different groups (n = 5), were immediately fixed in 2% glutaraldehyde and 2% paraformaldehyde in 0.1 M phosphate buffer (pH 7.4) for 2 h at 4°C. Following three washes in phosphate buffer, the brain tissues were post-fixed with 1% osmium tetroxide on ice for 2 h and washed three times, all in phosphate buffer. The tissues were then embedded in Epon 812 after dehydration in ethanol and propylene oxide series. Polymerization was conducted with pure resin at 70°C for 24 h. Ultrathin sections (~70 nm) were obtained with a model MT-X ultramicrotome (RMC, Tucson, AZ) and collected on 100 mesh copper grids. After staining with uranyl acetate and lead citrate, the sections were visualized using JEM-1400 Plus TEM at 120 kV (JEOL, Japan).

### Measurements for number and size of SVs

Samples were immediately prepared with control mice (n = 5) and RF-EMF exposed mice (n = 5). We generated images of 4–6 synapses per mouse and counted synaptic vesicles (SVs) in 21 and 24 synapses (4-week control and RF-EMF exposed group) and 21 and 24 synapses (12-week control and RF-EMF exposed group).

In addition, the area of synaptic vesicles (SVs) in all pre-synapse used for counting SV was measured. However, we simply selected only the SV membrane which was clearly distinguished and then diameter of selected SVs (4-week control 1769/ RF-EMF 1910 SVs and 12-week control 1091/ RF-EMF 1739 SVs) was measured.

The number of SVs per unit area (μm^2^) was obtained following the instructions; 1) Firstly, the number of pixels per 1μm length was calculated by dividing the number of pixels of the acquired the image by the length of the scale bar (5μm) using the 'ImageJ' program. 2) Next, the pre-synapse area was calculated in the image using the 'ImageJ' program, and then calculated the number of pixels only of the cytoplasm, excluding the area of the nucleus or mitochondria. The area (μm^2^) of pre-synapse was calculated by substituting the number of pixels per 1μm length obtained above. 3) Finally, the total number of SV in the pre-synapse counted using the cell counter function of 'ImageJ' program, divide by the pre-synapse area (μm^2^) and obtain SV number per 1 μm^2^.

The cross-sectional area of SVs (nm^2^) was obtained following the instructions; the size of synaptic vesicle (SV) was calculated by measuring the diameter (pixels) of each SV by drawing a line across the SV inner membrane using the 'ImageJ' program and the radius (pixels) of each SV was calculated (pixels). The pixel value was then calculated based on the scale bar to obtain the actual size value (nm). SV was assumed to be a circle, and the area [area (nm^2^) = (radius)^2^ (π = 3.14)] of each SV was calculated and the average value was measured. Its value is cross-sectional area of SVs (nm^2^).

### Electron tomography with Bio-HVEM and 3 D reconstruction

The tissue samples of the cerebral cortex from both, sham- and RF-EMF exposed mice (n = 5) were sectioned (500 nm thick) for 3D electron tomography of the synapses. The sections were placed on the 100 mesh copper grid, which was placed on a double tilting holder, and zero-loss images were viewed using an in-column omega filter equipped Bio-HVEM at 1,000 kV (JEM-1000BEF, JEOL, Tokyo, Japan). The region of interest was selected and the sample was tilted from +60° to -60° with 1° increments and a total of 121 tilt images were recorded using TEM Recorder software (JEOL System Technology Co., LTD., Tokyo, Japan). The digitized tilt series images were aligned and tomographically reconstructed using Composer and Visualizer-Kai software (TEMography.com, System in Frontiers Inc., Tokyo, Japan) with no fiducial markers. Subsequently, virtual slices were extracted from the reconstructed 3D tomogram, and the boundaries of the region of interest that were visible in each tomographic slice were traced as contours overlaid on the image. Object surface rendering and 3D volume modeling were performed using the AMIRA software (FEI, Hillsboro, OR).

### Reverse transcription and quantitative real-time PCR

Total RNA was purified using TRIzol reagent (Thermo Fisher Scientific, Pittsburgh, PA) from the cerebral cortex of mice (n = 10). RNA was reverse transcribed to cDNA using MMLV reverse transcriptase (Bioneer, Daejeon, South Korea) and an oligo-d(T)18 primer. Quantitative real time PCR (qRT-PCR) was carried out using Rotor Gene SYBR Green supermix Kit (QIAgen, Hilden, Germany) and fluorescence was measured using Rotor-gene PCR Cycler (QIAgen, Hilden, Germany). Glyceraldehyde 3-phosphate dehydrogenase (GAPDH) was used as a housekeeping gene. The primers were synthesized by Bioneer. The sequences for forward and reverse Syn primers were as follows: Syn I F: 5´- CAGGGTCAAGGCCGCCAGTC-3´and R: 5´-CACATCCTGGCTGGGTTTCTG-3´; Syn II F: 5´-AGGGGAGAAATTCCCAC-3´ and R: 5´-CCCAGAGCTTGTACCG-3´; Syn III F: 5´-CCAACAG-CGACTCTCG-3´ and R: 5´-GGTTGCGGATTGTCTC-3´ [20]. GAPDH primer was purchased from QIAgen. Three biologically independent experiments were performed and each PCR reaction was carried out in triplicate. The relative levels of specific mRNA were calculated by normalizing to the expression of GAPDH using the 2^-ΔΔCt^ method. Additionally, the expression values of the RF-EMF exposed groups were normalized to those of the sham-exposed group.

### Immunoblotting analysis

The cerebral cortex dissected from the brain of sham-exposed mice (n = 10) or RF-EMF exposed mice (n = 10) was lysed with RIPA buffer (ATTO, Tokyo, Japan) supplemented with protease and phosphatase inhibitor cocktail (ATTO, Tokyo, Japan). Whole lysates were then homogenized in ice-cold buffer and sonicated briefly. Protein concentrations were measured using a Bio-Rad DCTM protein assay (Bio-Rad, Hercules, CA) and total proteins (20–50 μg) were separated using electrophoresis in a 10% sodium dodecyl sulfate-polyacrylamide gel and transferred using transfer buffer to a polyvinylidene difluoride (PVDF) transfer membrane (ATTO, Tokyo, Japan). Syn I, Syn II, and α-tubulin were detected in the membranes using anti-synapsin I antibody (1:1000, Abcam #ab64581), anti-synapsin II antibody (1:3000, Abcam #ab76494) and anti-α-tubulin (1:3000, Santa Cruz #sc-23948). The protein bands were visualized using Odyssey infrared imaging system (Li-Cor Biosciences, Lincoln, NE). The intensity of each band was quantified, and was normalized using α-tubulin as an internal loading control.

### Statistical analysis

Data are presented as means ± SEM. The n values represent the number of animals used in experiments. The statistical significance of the data was assessed using Student’s *t*-test with probability values. Significance was defined as follows: *p<0.05, **p<0.01, ***p<0.001, ****p<0.0001. All statistical analyses were performed using used GraphPad Prism 4 program (GraphPad Software, Inc., La Jolla, CA).

## Results

### The number of SVs was decreased at presynaptic terminals of cortical neurons after RF-EMF exposure

**Fig 1:**
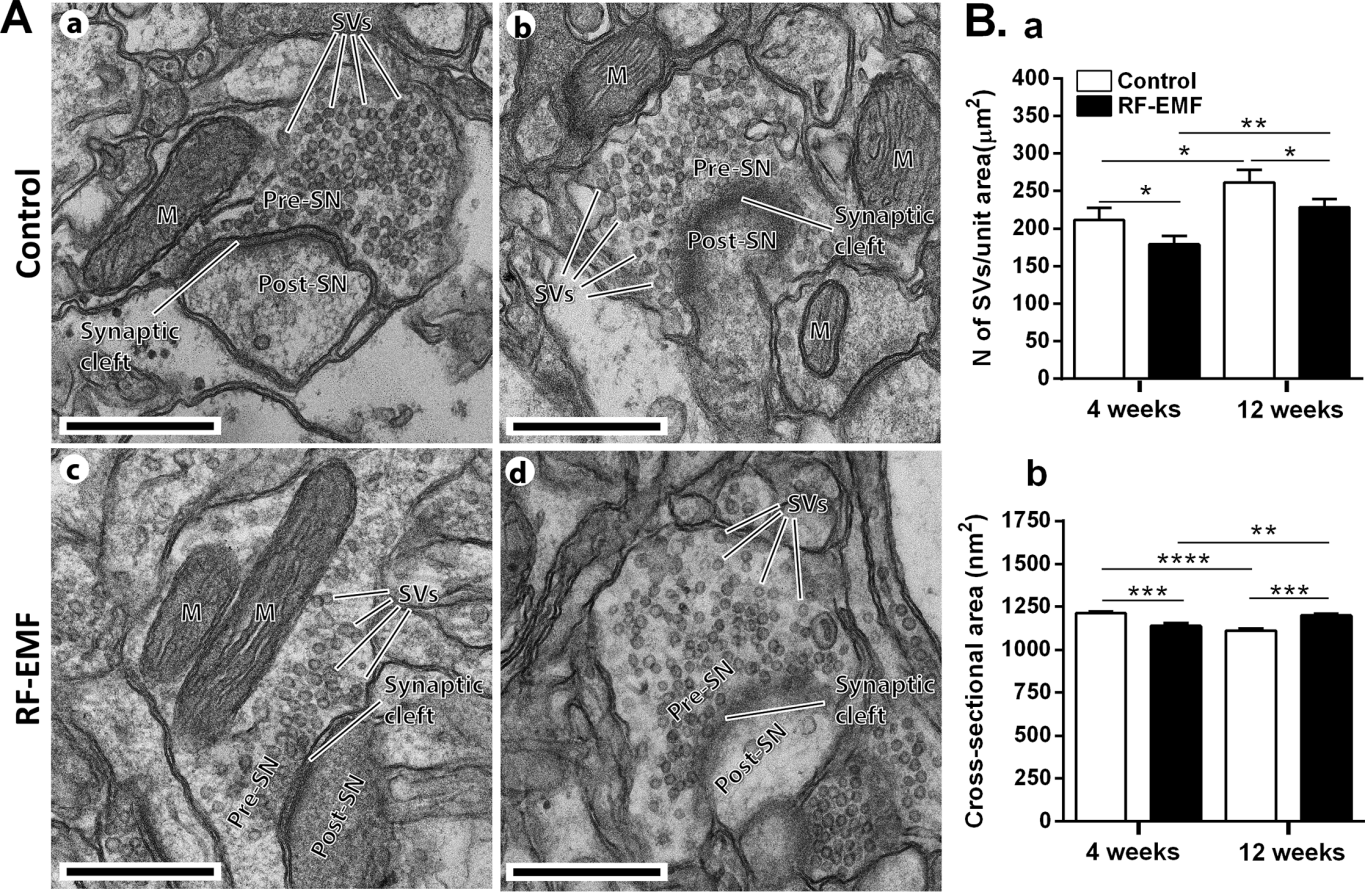
Ultrastructure of synaptic vesicles in the cerebral cortex of mice after 835 MHz radiofrequency (RF) exposure. (**A**) Representative TEM micrographs of the synapse region in the cerebral cortex were acquired from 12 week sham exposed (**a** and **b**) and RF-EMF exposed mice (**c** and **d**). **M**, mitochondria; **Pre-SN**, pre synaptic neuron; **Post-SN**, post synaptic neuron; **SVs**, synaptic vesicles; Size bars: 500 nm. Comparisons of synaptic vesicle number (**B**a, SVs per unit area (mm^2^)) and size (**B**b, the cross-section area (nm^2^)) at the presynaptic terminals in the cerebral cortex between sham-control and RF-EMF exposed mice for 4- and 12-week periods. Each bar represents the mean ± SEM. Statistical significance was evaluated using Student's t-test, *p <0.05, **p<0.01, ***p<0.001, ****p<0.0001.

To investigate whether RF-EMF exposure has an influence on the SVs of cortical neurons, we first analyzed the ultrastructural TEM images of the SVs in presynaptic terminals of cerebral cortical neurons of mice after 835 MHz RF-EMF exposure for 4 or 12 weeks. Fig 1A illustrates the images of representative synaptic neurons in the cerebral cortex of sham exposed (a and b) and RF-EMF exposed mice (c and d) with 12 weeks of exposure. To statistically analyze the alterations of SVs, randomly chosen EM images of synaptic boutons of the cerebral cortex neurons, 23 sham and 32 RF-EMF in the 4-week exposure group, and 21 sham and 24 RF-EMF in the 12-week exposure group, were used. We calculated the total number of SVs in a unit area (μm^2^) of the presynaptic terminals. The results indicated that the number of SVs in a unit area of presynaptic terminals was significantly decreased in the 4 weeks RF-EMF exposed group (179.3 ± 11.24) by comparing with the control group (211.6 ± 15.85). In parallel, the number of SVs in the 12 week RF-EMF exposure group (228.3 ± 10.78) was also significantly decreased by comparing with control group (261.9 ± 16.43) (Fig 1Ba). Of interest, in comparison between the control groups of 4 and 12 weeks, the number of SVs was significantly increased by 20% in 12 week exposed group. However, the size of the SVs (cross-sectional area; nm2) was decreased about 16% of those with sham-exposure (1213 ± 10.89) after the 4 week exposure to RF-EMF (1138 ± 16.92) but increased about 10% of those with sham-exposure (1110 ± 13.76) after 12 weeks of exposure to RF-EMF (1200 ± 11.60) (Fig 1Bb).

The TEM analysis indicated that RF-EMF induces alterations of SVs in the presynaptic nerve terminals in the cerebral cortex particularly, the number of SVs was slightly decreased after RF-EMFs exposure but the size of SVs was slightly increased after 12 weeks of RF-EMF exposure.

### The number of SVs was decreased at the synaptic terminals of cortical neurons after RF-EMF exposure in 3D model construction with Bio-HVEM

**Fig 2:**
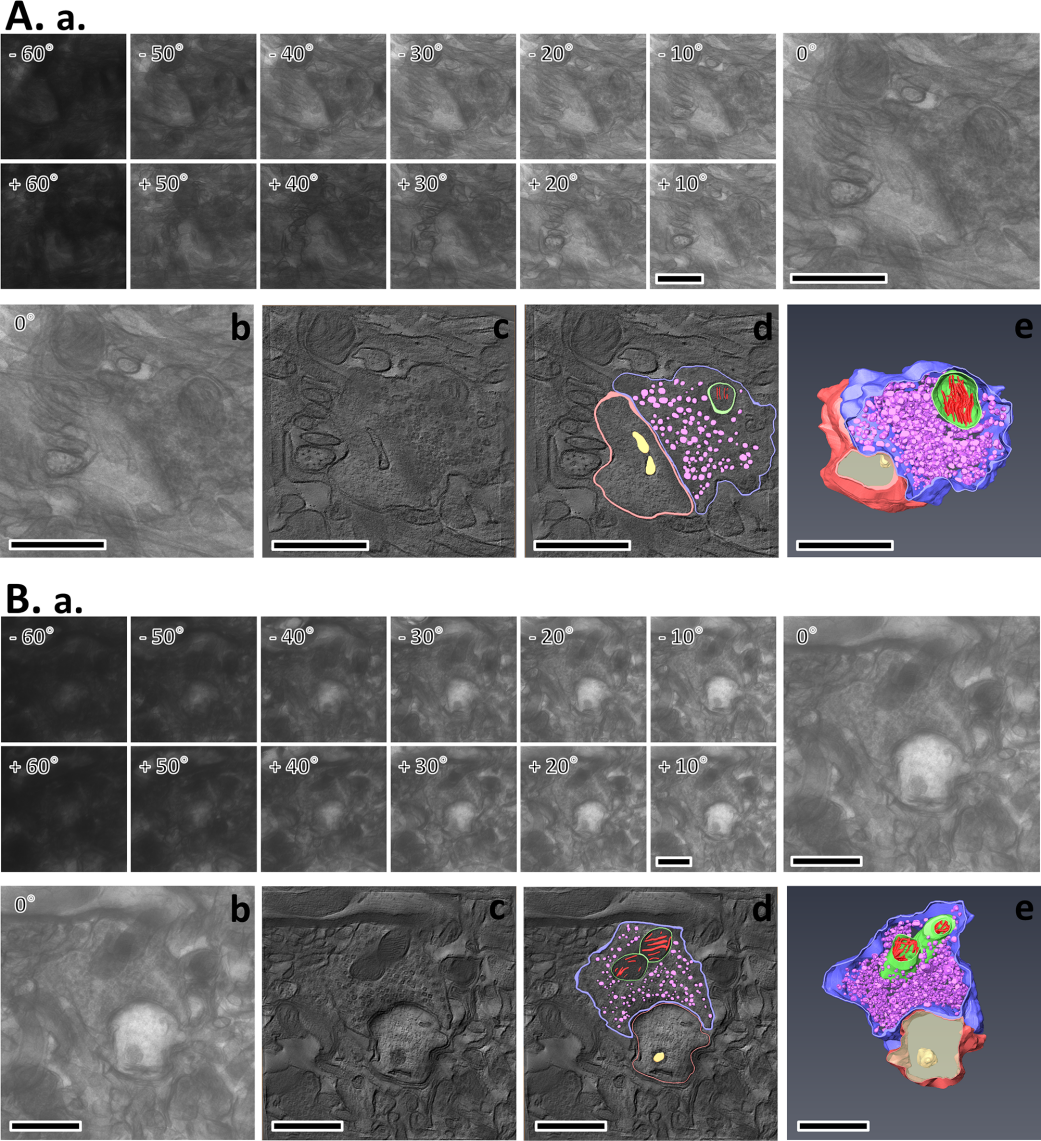
Electron tomography of presynaptic terminals in mice cerebral cortex following 12-weeks of RF-EMF exposure. Electron tomography process from two dimensional (2D) tilt series imaging to three dimensional (3D) modeling in the cerebral cortex of sham-exposed mouse (**A**) and RF-EMF exposed mouse (**B**). The tilt series of synapse image containing 121 images were recorded over a tilt range of -60° to +60°, with an interval of 1° using Bio-HVEM (**Aa and Ba**); the 0° reference image with Bio-HVEM (**Ab and Bb**); the virtual digital slice extracted from the 3D tomogram which was generated by alignment of tilt series images using Composer (**Ac and Bc**); boundaries of the region of interest that were visible in each tomographic slice were traced as contours overlaid on the image by AMIRA (FEI) (**Ad and Bd**); tomographic slices of surface of objects, including SVs, stacked to generate 3D models using AMIRA (**Ae and Be**). The SV membranes in pre-synaptic terminals are represented in violet color. Size bars: 1 μm.

**Fig 3:**
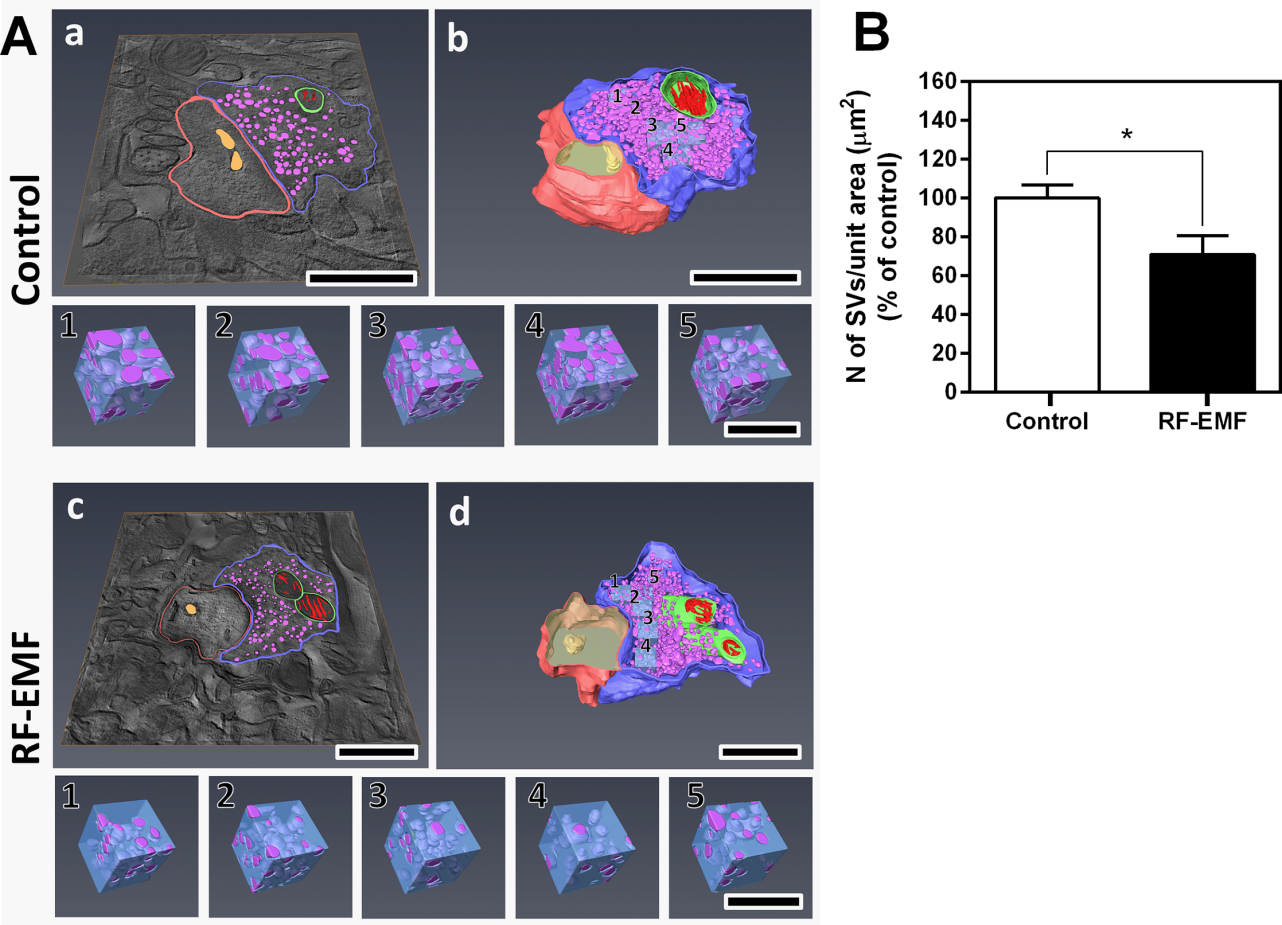
3D electron tomographic analysis of synaptic vesicle number at the presynaptic terminal in cerebral cortex after sham and 12-week RF-EMF exposure. The five 190 nm^3^ cubes were randomly extracted from the pre-synapse 3D model of sham-exposed (**A Control**) and RF-EMF (**A RF-EMF**) exposed condition, respectively. Rendering of tomographic slice (**Aa and c**), 3D model with cube (**Ab and d**), Cubes (**Control 1–5 and RF-EMF 1–5**). Size bars: 1 μm (a—d), 200 nm (cube 1–5). The SVs in each cube were counted and statistically analyzed. Each bar represents the mean ± SEM. Statistical significance was evaluated using Student's t-TEST: *p <0.05.

To further confirm whether RF-EMF exposure leads to a reduction in the number of SVs at the presynaptic terminals in the cerebral cortex, we investigated the ultrastructure of SVs in three-dimensions using Bio-HVEM and three-dimensional (3D) electron tomography analysis (Fig 2 and Fig 3). In this experiment, we determined the number of SVs in the 12-week exposed groups. Similar sized synapses were randomly chosen from the sham-exposed and RF-EMF exposed mice cortex and a 3D model of synaptic terminals and SVs was generated by sequential tilt series imaging from each tilt angle (Fig 2Aa and 2Ba), alignment and tomogram generation (Fig 2Ab, 2Ac, 2Bb and 2Bc), and rendering and 3D model construction (Fig 2Ad, 2Ae, 2Bd and 2Be). The completed 3D model of synapse, as shown in Fig 2D and 2E (rendering and 3D model) was used in Fig 3Aa abd 3Ac (rendering of tomographic slice) and A b, d (3D model with cubes) for the analysis of SV number. To count the SVs of cortical neurons, a cube of appropriate size was determined according to the shape of the synapse of the 3 D model, and five equal-sized cubes (190 nm^3^) were randomly inserted (Fig 3Ab and 3Ad) and extracted from SVs from the sham-exposed (Fig 3A Control 1–5) and RF-EMF exposed synapses (Fig 3A RF-EMF 1–5). The results number count of the SVs in each extracted cube showed that the number of SVs in the unit cubic volume of presynaptic terminals was decreased to 70.78 ± 9.94 percent of wild-type SV number after 12 weeks of exposure to RF-EMF similar to the two-dimensional (2D) TEM analysis data (Fig 3B). Altogether, 3D electron tomography analysis additionally indicated that RF-EMF exposure for 12 weeks could decrease the number of SVs in presynaptic terminals of the cerebral cortex.

### Decrease in the expression levels of Syns in the cortical neurons in response to RF-EMF exposure

**Fig 4:**
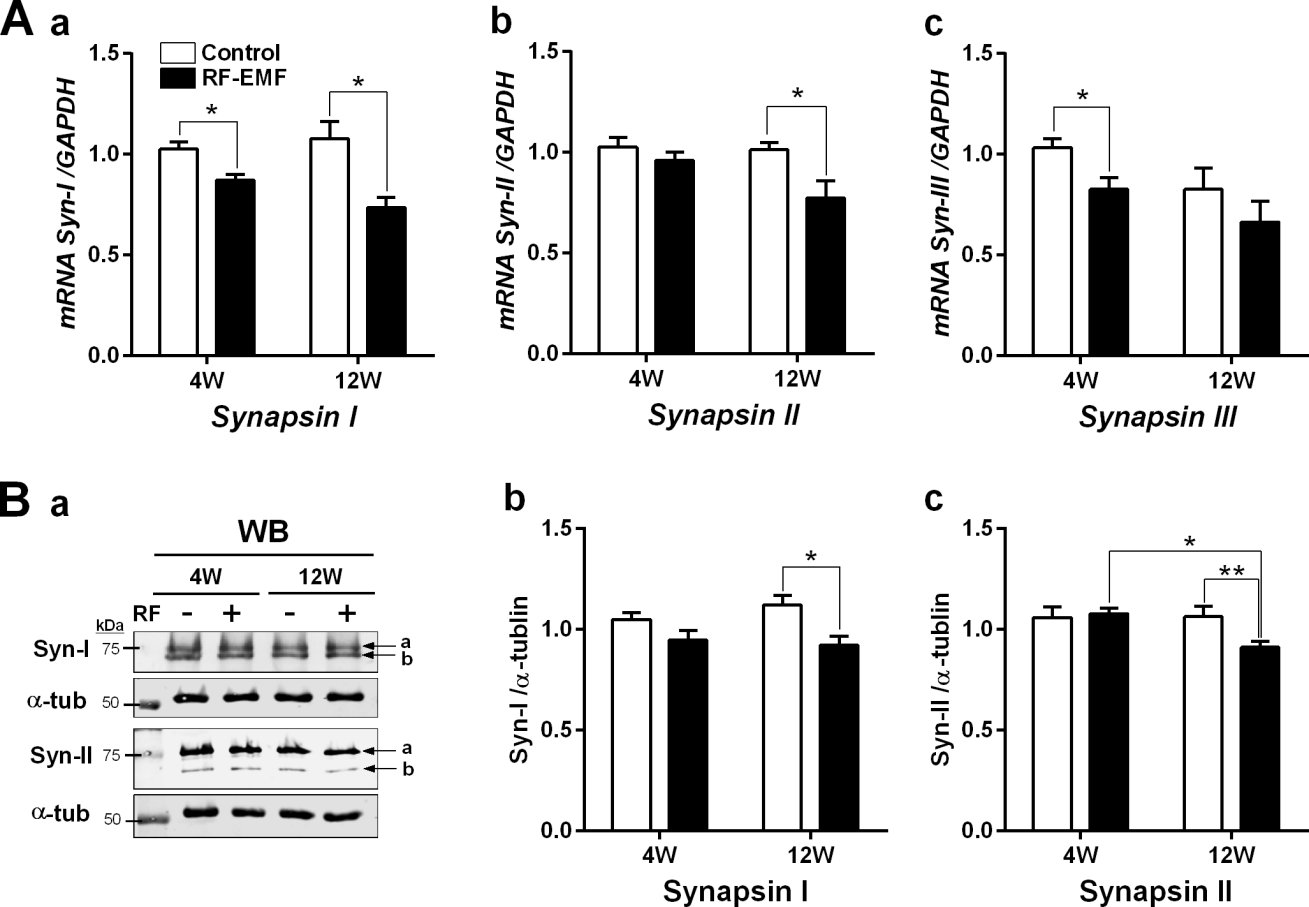
Expression levels of synapsins in the cerebral cortex of mice following RF-EMF exposure for 4 or 12 weeks. **(A)** Cortical total RNA extracted from sham and RF-EMF-exposed mice were analyzed for the expression level of synapsins I/II/III using quantitative real-time PCR (qRT-PCR). The relative mRNA levels of synapsins I/II/III (**a-c**) as calculated by normalizing to expression of GAPDH with the 2^-ΔΔCt^ method (n = 10). Each bar represents the mean ± SEM. Statistical significance was evaluated using two-tailed unpaired Student t-test (*p<0.05). **(B)** Levels of synapsin I/II proteins in the cerebral cortex of mice after RF-EMF exposure for 4 weeks (4W) or 12 weeks (12W). Representative immunoblots of synapsin I and synapsin II (**a**). The intensity of the bands was quantified **(b** and **c)**. The protein level of synapsin I/II was normalized to α-tubulin. Each bar shows mean with SEM. Statistical significance was evaluated using two-tailed unpaired Student's t-test: *p<0.05, **p<0.01.

As Syn is a key regulator of storage and mobilization of SVs at synaptic terminals, we further investigated the expressional changes of genes and proteins after RF-EMF exposure using qRT-PCR and immunoblot. The results of qRT-PCR indicated that transcriptional levels of Syns I/II/III were gradually deceased in the cerebral cortex of mice after RF-EMF exposure (Fig 4A). The expression level of Syn I mRNA in the cerebral cortex of mice was significantly reduced by about 15% after 4-week exposure and by around 25% in the 12-week exposure group compared with that in sham-exposed normal group (Fig 4Aa). However, the levels of Syn II and Syn III showed significant decreases in the mice cerebral cortex only after 12-week RF-EMF exposure (Fig 4Ab) and 4-week RF-EMF exposure (Fig 4Ac), respectively. Overall, Syns I/II/III transcript levels were decreased in a time-dependent manner with RF-EMF exposure (Fig 4A). To validate the qRT-PCR result, the expression levels of Syn I and Syn II proteins were determined using Western blotting with anti-synapsin-I and anti-synapsin-II antibodies in the cerebral cortical lysates. Both the antibodies can detect both a and b subunits of synapsin. The protein levels of both, Syn I and Syn II significantly decreased in the cerebral cortex after RF-EMF exposure (Fig 4B). These data indicated that the expression level of Syn genes and proteins were significantly down-regulated in cerebral cortex during the RF-EMF exposure in an exposure-time dependent manner.

## Discussion

Our study showed that the number of SVs at synaptic terminals in the cerebral cortex can be affected by long term exposure of 835 MHz RF-EMF (4.0 W/kg SAR for 5 h/day for 4–12 weeks). In this study, we obtained 2D TEM images for presynaptic terminals using the conventional TEM (Fig 1) and further examined the 3D images of presynaptic boutons of cerebral cortical neurons using Bio-HVEM (Fig 2). The 2D TEM image analysis showed that the number of SVs was significantly decreased in the presynaptic terminals of cerebral cortical neurons after both, 4 and 12 weeks of RF-EMF exposure. In addition, the density of the SVs was reduced by more than 15% as evaluated with the 3D model of presynaptic boutons obtained using Bio-HVEM. The measurements using Bio-HVEM involved counting the number of SVs contained in five independent cubes of the same size (Fig 3) inserted in the images of sham and RF-EMF exposed mice groups. Therefore, our results indicated that exposure to RF-EMF causes a decrease of the number of SVs in presynaptic terminals at cerebral cortical neurons and the present study suggested that the Bio-HVEM approach can be also a reliable and robust method for measure the number of SVs at synaptic terminals in 3D by overcoming the planar limit imposed by 2D images. So, the methods for the conventional TEM for 2D model as well as the Bio-HVEM for 3D model can be a reliable manner to measure the number of SVs efficiently.

Importantly, synapses are formed by inter-neuronal connections that implicate the transmission of electrical or chemical signals from one neuron to another. SVs, small spherical organelles located at presynaptic nerve terminals that mainly regulate neurotransmitter storage, release, and secretion, which are accomplished by association with various synaptic proteins including Syns [21]. To determine whether the numerical change of SVs is influenced by changes of other factors after RF-EMF exposure, we examined the expression level of the Syns. As reported previously, various synaptic proteins may associate with and regulate the storage and mobilization of SVs but we focused on the Syns, which are one of the key synaptic vesicle proteins [21]. Syns are a family of neuronal SV-associated proteins that have long been implicated in the regulation of neurotransmitter release at synapses [18]. The expression of Syns I/II genes and Syns I/II proteins were significantly decreased in cerebral cortical neurons of 12 weeks of RF-EMF exposure in the mice (Fig 4). However, Syn III transcriptional levels were difficult to detect due to low basal levels of Syn III in adult brain although it is highly expressed in early developmental stages of mice brain [22]. The tendency of reduction of synapsins level post-12 week exposure to RF-EMF may indicate that Syns I/II could be further reduced if exposed to RF-EMF for a longer period of stronger stimulation than used in this study. Thus, the numerical alteration of SVs may be attributed to change in synaptic proteins such as Syns in cerebral cortical neurons of mice after RF-EMF exposure. In addition, it was confirmed with EM analysis that the size of SVs slightly but significantly increased after 12 weeks of RF-EMF exposure. This is consistent with findings that a deficiency of Syns I and/or II caused a decrease of the number of SVs in the specific region of mouse brain, but caused an increase of the size of SVs. Larger SV size may be a compensatory process for the reduced numbers of SVs [23–26].

The number of SVs in presynaptic nerve terminals cannot be a direct estimate of the amount of neurotransmitter but SVs function in the storage of neurotransmitters, therefore, the number of SVs is increased in proportion with the amount of stored neurotransmitter at synaptic terminals. Larger amounts of neurotransmitters in presynaptic terminals would require larger numbers of SVs to efficiently transfer neurotransmitters to postsynaptic regions.

Specifically, we focused on the cerebral cortex of mice on this study. Basically, the human cerebral cortex is an important brain area for processes related to sensory and cognitive functions [14, 27]. It can be divided into four areas, which function differently. However, our results analyzed cortical neurons as a whole, ignoring the four divisions of the cerebral cortex, and repeatedly obtained consistent results indicating that RF-EMF exposure leads to reduced numbers of SVs and decreased levels of Syns in the mice cerebral cortex.

To summarize, exposure to 835 MHz RF-EMF, 4 W/kg, 5 hours per day for 4 and 12 weeks leads to a decrease in the number of SVs at presynaptic terminals and to reduction in the levels of Syns I/II. Importantly, the reduced number of SVs may lead to alteration of neurotransmitters loading and release. In addition, decrease in synaptic vesicle proteins such as Syns I/II could lead to reduced numbers of SVs after RF-EMF exposure. Thus, neurotransmitter storage and transport by SVs may be functionally affected in the mice cortical neuron by RF-EMF exposure. Finally, these results may provide the insight that the reduction of neurotransmitters may cause neurological dysfunction in the cerebral cortex, which could further lead to neurobehavioral disorders.
